# Robotic-assisted resection of pediatric intracavitary lesions: expanding the boundaries of minimally invasive surgery

**DOI:** 10.1007/s00383-026-06560-x

**Published:** 2026-08-03

**Authors:** Alejandro O. Chara, Vikas S. Gupta, James L. Rogers, Roshan J. D’Cruz, Megan Stout, Brendan Frainey, Lauren Corona, Douglass B. Clayton, Harold N. Lovvorn, Irving J. Zamora

**Affiliations:** 1 Department of Surgery, Vanderbilt University Medical Center, 1211 Medical Center Drive, Nashville, TN USA; 2 Department of Pediatric Surgery, Monroe Carrell Jr. Children’s Hospital, 2200 Children’s Way, Nashville, TN USA; 3 Vanderbilt University School of Medicine, 2209 Garland Avenue, Nashville, TN USA; 4 Department of Pediatric Surgery, Medical College of Georgia at August University, 1120 15th Street, Augusta, GA USA; 5 Department of Pediatric Urology, Monroe Carrell Jr. Children’s Hospital, 1211 Medical Center Drive, Nashville, TN USA

**Keywords:** Robotic surgery, Pediatric surgical oncology, Pediatric masses, Urologic masses, Intracavitary masses, Minimally invasive surgery

## Abstract

**Purpose:**

Describe the implementation of robotic-assisted surgery for pediatric cysts and neoplasms and characterize perioperative outcomes.

**Methods:**

A retrospective review was performed of pediatric patients undergoing robotic-assisted resection of cysts or neoplasms at a quaternary children’s hospital from 2011 to 2026. Demographic, lesion, operative, and outcome data were analyzed.

**Results:**

Thirty-eight pediatric robotic cases were reviewed. Median age was 13 years (IQR 8.25–16). Eighteen intracavitary cysts and 20 neoplasms were resected. Operations for solid neoplasms consisted of thymectomies (*n* = 3), mediastinal teratoma excision, lung lobectomy, partial gastrectomy, left hepatectomy, distal pancreatectomy (*n* = 2), pancreatic uncinate resection, pancreaticoduodenectomy, nephrectomies (2 partial, 2 radical), adrenalectomy, periadrenal mass resection, ovarian cystectomy (*n* = 2), and radical cystoprostatectomy. Median EBL was 15 mL (IQR 5–25) and day of discharge 1 (IQR 0–2). The median opioid dose prescribed at discharge was 0.28 mg/kg oral morphine equivalents with duration of 1 (0–3) day. Thirty-one (82%) patients experienced no complications. Major complications occurred after pancreatic resections, including hemorrhagic pancreatitis following uncinate resection and delayed biliary stenosis with ductal leak after pancreaticoduodenectomy requiring revision 1.5 years later.

**Conclusion:**

Robotic-assisted surgery is a safe and feasible option for selected pediatric cysts and neoplasms, offering low blood loss, short hospitalization, and minimal postoperative opioid requirements.

## Introduction

Pediatric surgery has evolved toward less invasive, image-guided approaches for resecting complex cystic and solid tumors and meeting patients in the context of their disease when malignant [1–4]. Complete tumor removal is challenging in children due to size and anatomic constraints, the need for meticulous dissection, and the importance of minimizing long-term morbidity in growing patients [1, 5]. Over the past two decades, minimally invasive surgery (MIS), including laparoscopy and robotic-assisted techniques, have become more widely adopted in pediatric surgical care. MIS now accounts for up to 30–50% of diagnostic procedures and approximately 20% of curative resections [1, 6, 7]. However, conventional laparoscopy remains limited by two-dimensional visualization, restricted instrument dexterity, and ergonomic challenges, particularly in confined spaces or near critical vascular structures [8–10]. Robotic-assisted surgery addresses several of these limitations, and recent studies support its safety and feasibility in carefully selected pediatric patients [3, 11, 12].

Limited data exist on the use of robotic surgery for resection of pediatric cystic and solid lesions. Robotic-assisted surgery in this area derives from small retrospective cohort studies, expert opinion, or data extrapolated from adult studies [3, 11, 12]. The lack of randomized controlled trials and large cohort studies prevents the establishment of clear, evidence-based guidelines for the use of robotic-assisted surgery in intracavitary resections [10, 13]. In children compared to adults, the lower incidence of neoplastic diseases, larger tumor variety, and technical implications further complicate the development of multi-center randomized controlled trials. Therefore, investigation of the perioperative outcomes in this population is crucial to the progression of robotic-assisted surgery.

The objectives of this single-institution study were to: (1) examine the safety and feasibility of robotic-assisted resection of benign and malignant lesions in pediatric patients over a 14-year period; (2) describe perioperative outcomes, including operative time, conversion rates, postoperative complications, and length of stay; and (3) evaluate oncologic outcomes, including completeness of resection and recurrence rates for malignant lesions.

## Methods

### Data source and study population

This IRB-approved (#260477) descriptive single-center study queried patients from the Research Derivative institutional database, which is derived from the clinical system of this free-standing pediatric quaternary care center. Any missing data from the database were collected by accessing patient medical records via the hospital’s electronic medical system. Both inpatient and follow-up data were available via the same medical record.

We included all pediatric patients 19 years or younger undergoing robotic-assisted excision of either benign or malignant lesions. Patients were treated with the da Vinci SI^®^ surgical robot system (Intuitive Surgical; Sunnyvale, CA) between 2011 and 2021 and with the da Vinci XI^®^ between 2021 and 2026. Children who underwent robotic-assisted procedures for other indications, for example, those undergoing robotic interventions outside the abdomen or thoracic cavities, or those with lesions not resected robotically were excluded. Pediatric patients undergoing operations exclusively at the main adult hospital without pediatric surgery involvement were excluded.

### Variables of interest

Demographic information, including age, gender, and ethnicity, was collected for each patient. Diagnosis, cavitary location, use of preoperative regional anesthesia, estimated blood loss (EBL), operative service, operation duration, and conversion rate were evaluated. Final pathology classified lesions as benign or malignant. Cavitary location was classified as either abdominal or thoracic. Preoperative regional anesthesia included transversus abdominis plane (TAP) block, erector spinae plane (ESP) block, thoracic epidural catheter (TEC) placement, or pudendal blocks.

### Patient selection and case planning

Not all patients with resectable mass lesions are candidates for robotic-assisted surgery. Patient selection begins with multidisciplinary review involving pediatric surgeons, oncologists, and radiologists to assess tumor location, tissue planes, proximity to critical vascular and biliary structures, anticipated margin status, and the feasibility of a minimally invasive approach while maintaining oncologic principles [14–16]. Patients selected for robotic resection typically have well-circumscribed lesions, favorable dissection planes, limited vascular involvement, and anatomy that allows safe port placement and instrument triangulation. Additional considerations include tumor size relative to patient anatomy, specimen extraction requirements, prior surgical history, and the potential need for vascular reconstruction or multivisceral resection. Relative contraindications at our institution include extensive intravascular involvement, tumor burden that limits working space, inability to achieve an oncologically sound resection robotically, and the anticipated need for a large extraction incision.

As our robotic program has expanded to more complex pathology, we have developed a collaborative model with adult surgical oncology, hepatobiliary, colorectal, and thoracic specialists. These surgeons may participate in preoperative imaging review and operative planning, meet patients and families preoperatively, and provide intraoperative consultation or co-surgeon assistance when advanced dissection, vascular control, or organ-specific expertise is required. This approach broadens procedural capabilities while maintaining patient safety and oncologic rigor.

Once a patient is deemed a robotic surgery candidate, perioperative stakeholders, including nursing leadership, operating room scheduling and supply management, and regional and general anesthesia teams, are engaged to complete preparation. Perioperative planning including case timing, patient position, instruments required, regional anesthesia options, and anesthetic considerations (lines, Foley catheter, etc.) are discussed.

Most operations include two faculty surgeons and a surgical fellow. The surgical fellow typically operates on the console alongside one of the faculty surgeons. The second faculty surgeon is at bedside for instrument exchange, manual retraction/tissue manipulation, and ready in the event emergent conversion to open is necessary. In cases where intraoperative ultrasound is utilized, pediatric radiologists join for live interpretation in the operating room. Outcomes are tracked closely and reviewed in tumor board or conferences with the same pre- and peri-operative team.

### Postoperative outcomes

Postoperative pain control, morphine milligram equivalents (MME) at discharge, postoperative day (POD) at discharge, and postoperative surgical complications at any point were assessed. Postoperative pain was subjectively determined as “controlled” versus “uncontrolled” based on inpatient and outpatient notes. Opioids prescribed at discharge were converted to MME for objective comparison [17].

### Statistical analysis

Descriptive statistics were used to analyze the cohort and summarize measured variables. Continuous variables were assessed for distribution and are reported as medians with interquartile ranges (IQR, 25th–75th percentile). Categorical variables are presented as frequencies and percentages. No formal comparative testing was performed, as this study was designed as a feasibility and safety analysis without a predefined control group. All analyses were performed using Microsoft Excel (Microsoft 365, Microsoft Corporation, Redmond, WA, USA).

## Results

### Patient characteristics

**Fig. 1:**
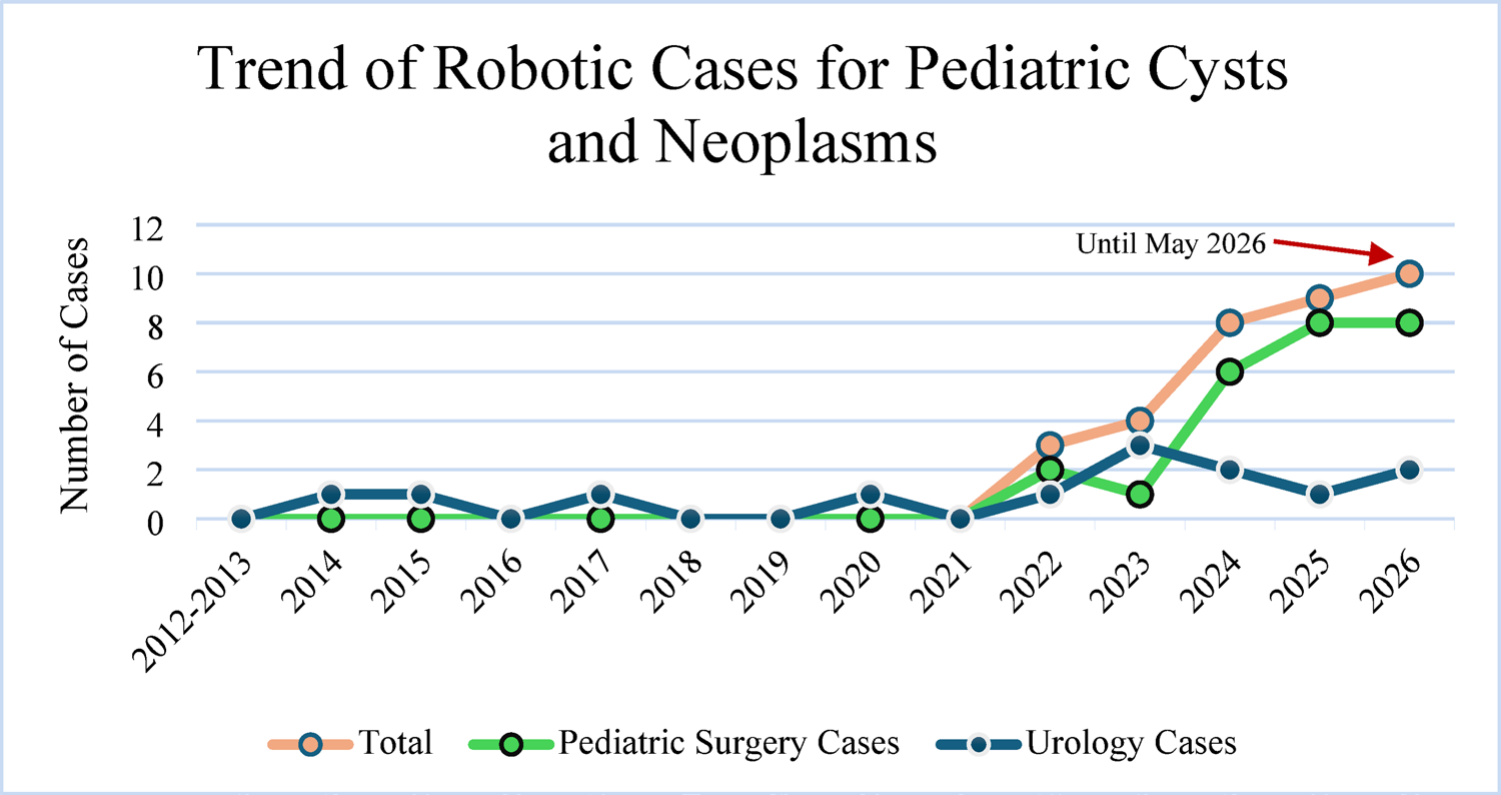
Trend in pediatric robotic-assisted cases for management of intracavitary cysts and neoplasms

Over the study period, a total of 38 cases were identified. The median age for our cohort was 11 years (5–15) for cysts and 13 years (11.25–16) for solid masses. Twenty-one of the 38 patients (55%) were female. Patients with a diagnosis of cysts had a median follow up of 63 days (36.75–351). Those patients with benign masses were followed up to 40.5 days (25.5–277.75) postoperatively while those with malignant masses were followed for 447 days (134–493). Table 1 provides additional demographic descriptors for our cohort. Figure 1 shows the trend of robotic-assisted surgery for these lesions since the implementation of the robot program at our institution (2011).

**Table 1:** Cohort description according to lesion type

	Benign solid masses (*n* = 12)	Malignant masses (*n* = 8)	Cysts (*n* = 18)
Female gender	6 (50%)	5 (62.5%)	10 (55.5%)
*Race/ethnicity*			
Non-Hispanic White	7 (58.3%)	6 (75%)	14 (77.8%)
Hispanic	1 (8.3%)	0 (0%)	0 (0%)
African American	4 (33.3%)	2 (25%)	4 (22.2%)
Median age at surgery (years)	14 (13–16.25)	10 (5.75–13.75)	11 (5–15)
Median weight at surgery (kg)	60.86 (50.38–82.82)	40.3 (30.8–66.88)	43.9 (20.1–81.65)
Median BMI at surgery (kg/m^2^)	22.5 (19.15–26.81)	20 (18.25–24.55)	17.6 (16.25–26.55)
*ASA status*			
One	0 (0%)	0 (0%)	0 (0%)
Two	4 (33.3%)	2 (25%)	16 (88.9%)
Three	8 (66.7%)	6 (75%)	2 (11.1%)
Four	0 (0%)	0 (0%)	0 (0%)
*Anatomical location*			
Thoracic	5 (41.7%)	0 (0%)	5 (27.8%)
Abdominal	7 (58.3%)	8 (100%)	13 (72.2%)

### Operative data

**Fig. 2:**
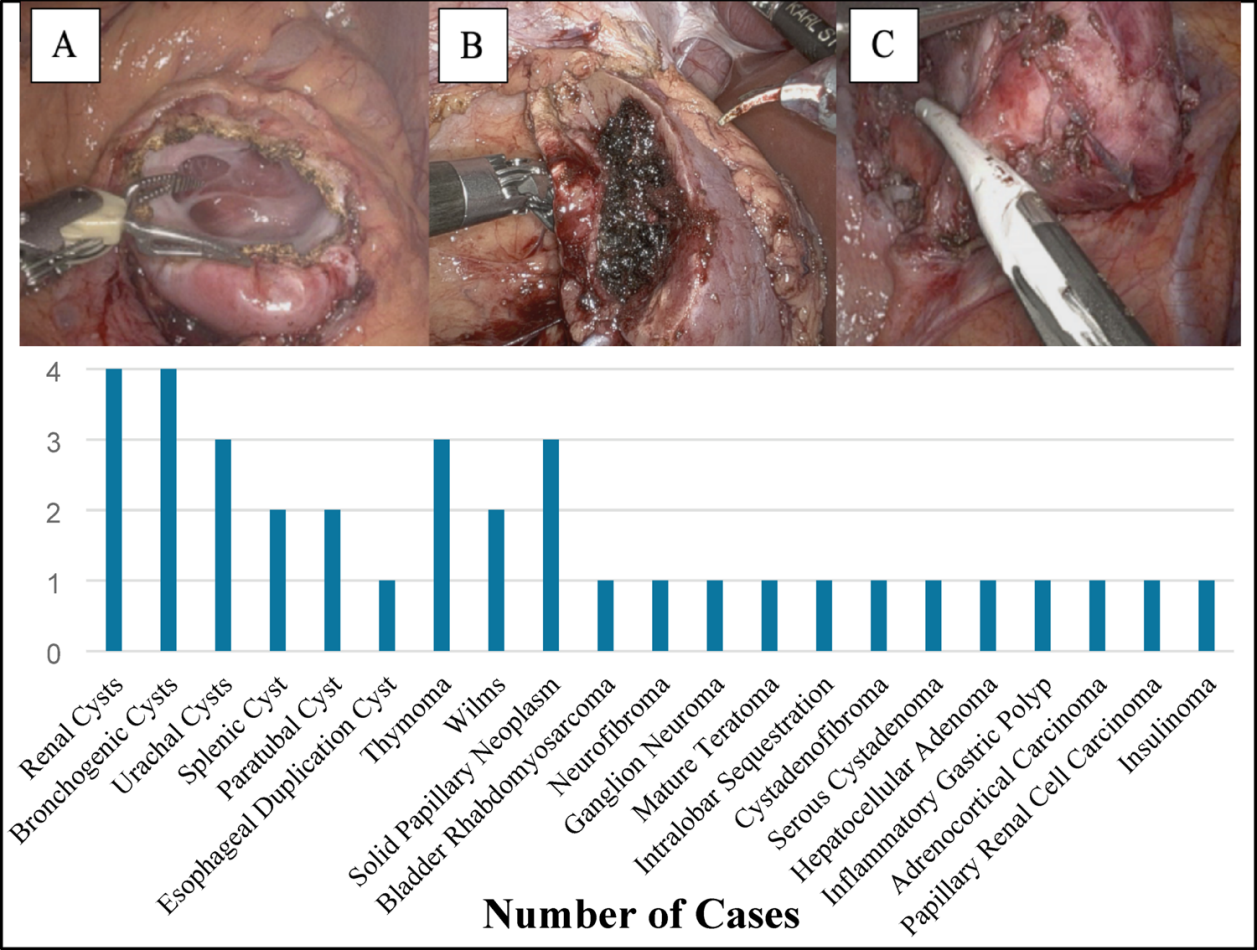
Number of robotic cases based on diagnosis. Intraoperative images depicting renal cyst decortication (**a**), partial nephrectomy for Wilms tumor (**b**), and resection of mediastinal mature teratoma (**c**)

The pediatric surgery service performed 25 (65.7%) of these cases; the pediatric urology service performed the remaining 13 cases. Twenty-eight (73.7%) of the mass-lesions were abdominal, while the remainder (*n* = 10) were thoracic. Twenty cases were completed for solid masses, of which 8 returned as malignant on final pathology. Operations for solid neoplasms consisted of thymectomies (*n* = 3), mediastinal teratoma excision, lung lobectomy, partial gastrectomy, left hepatectomy, distal pancreatectomy (*n* = 2), pancreatic uncinate resection, pancreaticoduodenectomy, nephrectomies (*n* = 2 partial, *n* = 2 radical), adrenalectomy, periadrenal mass excision, ovarian cystectomy (*n* = 2), and radical cystoprostatectomy. The remaining cases consisted of various cysts (Fig. 2).

Preoperative regional anesthesia was used in 29 (76.3%) cases. TAP blocks were used in 19 patients. An additional rectus sheath block was performed on seven of these patients. Seven patients received an ESP block and two patients received a TEC as part of their pain management. One patient received a pudendal block before surgery.

Cyst excisions had a median EBL of 7.5mL (0.5–23.75) and a median operative time of 171.5 min (124.25–219.5). Median EBL for solid mass resections was similarly minimal at 15mL (10–27.5) and with a slight longer median operative time at 225 min (145.75–292.25). The operative course in all but one case was uneventful. A robotic-assisted thoracoscopy to resect a left bronchogenic cyst was converted to open. During dissection, a small rent in the membranous portion of a left segmental bronchus to the lower lobe was detected after separating the common wall. Robotic repair was attempted but unsuccessful due to space constraints; therefore, a muscle-sparing thoracotomy was performed for repair.

**Fig. 3:**
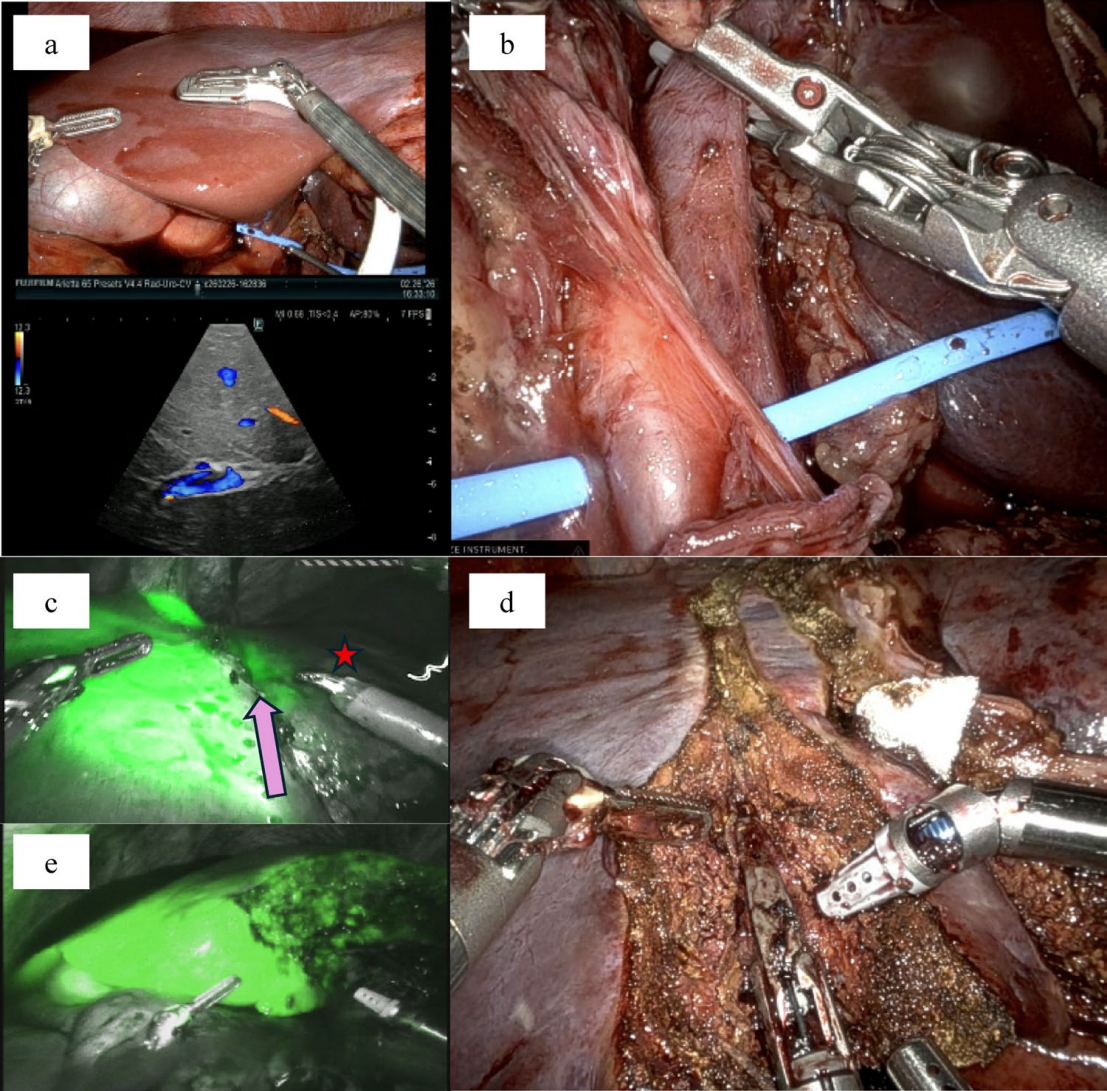
Hepatectomy case example of surgical adjuncts in robotic-assisted resection of mass lesions. **a** Intraoperative ultrasound delivers precise vascular identification of the portal system. **b** The left portal vein (yellow arrow) is then circumferentially controlled and clipped. **c** Following inflow isolation, intravenous indocyanine green (ICG) delineates a clear line of demarcation for transection. **d** Parenchymal transection proceeds with minimal blood loss along the previously identified margin. **e** Confirmation of adequate perfusion to remaining liver segments with ICG after parenchymal transection

For all cases, histologic diagnosis was determined based on final pathology. R0 resections were performed for all malignant lesions and there were no instances of tumor spillage intraoperatively. Table 2 shows detailed information on the final diagnosis and operative information. Figure 3 showcases intraoperative images and adjunct techniques available with the robotic platform.

**Table 2:** Intraoperative and postoperative objective data

Patient #	Age (yr)	Sex	Procedure	Diagnosis	EBL (mL)	DC Day	MME at DC (mg/kg)
1	2	M	Radical cystoprostatectomy w/ Ileal conduit	Embroyonal rhabdomyosarcoma (group III, stage III) of the bladder	35	8	0
2	3	M	Utricle cyst excision	Utricle Cyst	0	0	0
3	3	F	Thoracic mass resection	Right bronchogenic duplication cyst	10	1	1.68
4	4	M	Renal cystectomy	Left simple cyst	5	1	0.60
5	4	F	Renal cystectomy	Large left simple renal cyst	2	0	0
6	4	M	Urachal cyst excision	Urachal cyst	5	1	0.62
7	5	M	Radical nephrectomy with partial uureterectomy	Wilms (stage III)	5	4	0.45
8	6	F	Partial nephrectomy	Wilms tumor (stage 1, recurrence)	42	2	0.56
9	8	F	Thoracic Mass Resection	L bronchogenic cyst	33	2	0
10	8	F	Periadrenal Mass Resection	Stage 1 adrenocortical carcinoma	5	1	0.30
11	9	M	Radical nephrectomy	Neurofibroma	25	2	0
12	10	M	Mediastinal mass resection	Esophageal duplication cyst	20	1	0.66
13	10	F	Thoracic mass resection	Bronchogenic cyst	0	0	0
14	10	M	Urachal cyst excision	Urachal cyst	0	0	0.27
15	12	F	Thoracic mass resection	Bronchogenic cyst	25	1	0
16	12	F	Distal pancreatectomy	Solid pseudopapillary neoplasm	15	2	0.63
17	13	M	Mediastinal mass resection	Right mature teratoma	50	2	0
18	13	F	Ovarian cystectomy	Cystadenofibroma	5	0	0
19	13	F	Ovarian cystectomy	Serous cystadenoma	10	0	0
20	13	M	Distal pancreatectomy	G2 well-differentiated insulinoma	10	4	0
21	13	M	Partial splenectomy	Simple splenic cyst	17	1	0.69
22	13	F	Pancreaticoduodenectomy	Solid pseudopapillary neoplasm	15	4	0.22
23	14	M	Left hepatectomy	Hepatocellular adenoma	160	2	0.46
24	14	F	Renal cystectomy	Benign cystic lesion	50	1	0
25	14	F	Thymectomy	Thymoma	10	0	0.35
26	15	M	Urachal cyst excision	Urachal cyst	0	0	0.37
27	15	M	Renal cystectomy	Renal cyst decortication	5	0	0.59
28	16	F	Right lower lobectomy	Intralobar bronchopulmonary sequestration	20	1	0.32
29	16	M	Thymectomy	Thymoma	15	1	0.55
30	16	F	Ovarian cystectomy	Benign paratubal cyst	0	1	0
31	16	F	Ovarian cystectomy	Right paratubal ovarian cyst	20	0	0.45
32	16	M	Pancreatic uncinate process resection	Solid pseudopapillary neoplasm	50	1	0.35
33	16	F	Partial nephrectomy	Papillary renal cell carcinoma (T1a)	25	1	0
34	17	F	Renal cystectomy	Left renal cyst	65	0	0
35	17	M	Thymectomy	Thymoma	15	1	0
36	18	F	Splenic cystectomy	Large epithelial cyst	30	1	0.60
37	19	F	Adrenalectomy	Ganglion neuroma	17	2	0.67
38	19	F	Partial gastrectomy	Inflammatory fibroid polyp	10	13	0

*DC*  discharge, *MME* morphine miliequivalent

### Postoperative outcomes

The median postoperative day of discharge for cyst excisions was day 1 (IQR 0–1). Following solid mass resections, patients remained in the hospital for a median of 2 days (1–2.5). Eleven patients were discharged on the date of surgery, which included six patients undergoing urological cyst excision, three ovarian cysts, one bronchogenic cyst excision, and one thymoma. Based on daily progress and outpatient notes, pain was well controlled with multimodal pain regimen in all but one patient who underwent a robotic resection of a solid pseudopapillary neoplasm (SPN) from the pancreatic uncinate process. Seventeen (44.7%) patients did not require opioids at the time of discharge. This included 8 patients who underwent solid mass resections. Median duration of opioids at discharge for patients following robotic cyst excisions was 0.5 days (0–3) with a median morphine equivalent dosage of 0.13 mg/kg (0–0.53). Patients who had solid mass resections had a median duration of opioids of 1 day (0–2.25) with a median morphine equivalent dosage of 0.31 mg/kg (0–0.59). No additional opioids were prescribed after discharge.

Thirty-one (82%) patients had no postoperative complications and have remained free of recurrent disease, whether benign or malignant. Five patients required either surgical or interventional radiology procedures postoperatively. One patient required intermittent dilation for postoperative urethral stricture following utricle cyst excision. Another patient had recurrence of renal cyst but was treated with aspiration and sclerotherapy. The patient who underwent a pancreaticoduodenectomy developed biliary narrowing 1.5 years postoperatively and required robotic Roux-en-Y reconstruction. The patient who underwent pancreatic uncinate process resections developed hemorrhagic pancreatitis on POD3 and subsequent rim-enhancing fluid collection one month postoperatively. This was managed with IR drainage and eventual ERCP with stent placement. Additionally, one patient had recurrent disease from rhabdomyosarcoma 5 months after initial resection, leading to a closed-loop obstruction. Details regarding postoperative complications and Clavien-Dindo classification can be found in Table 3.

**Table 3:** Clavien-Dindo classification and postoperative complications

Patient	Clavien-Dindo classification	Complication
1	IVa	Ileus POD 3; tumor recurrent at 5 months; closed loop obstruction requiring laparotomy at 8 months; metastatic disease and death at 15 months
2	II	Urethral stricture
5	IIIb	Recurrent cyst requiring aspiration and sclerotherapy
9	II	Conversion to open due to small bronchial hole was noticed on the subsegmental bronchi on the left lower lobe
16	I	Grade B pancreatic leak treated with antibiotics
22	IIIb	Narrowing of biliary anastomosis 1.5yrs following robotic pancreaticoduodenectomy requiring IR drain and robotic Roux-en-Y HJ reconstruction
32	IIIb	Hemorrhagic pancreatitis on POD 3 requiring 2 IR drains following pancreatic uncinate process resection. Developed rim-enhancing fluid collection 1 month post-op requiring another IR drain and ERCP with stent placement

## Discussion

Minimally invasive surgery (MIS) for the definitive management of intracavitary cysts and neoplasms in children has evolved substantially over the past three decades and now includes robotic technology. Advances in visualization, instrument articulation, and surgeon ergonomics have facilitated broader adoption of robotic surgery for complex resections in confined anatomical spaces [18]. In our experience, robotic-assisted surgery was safely implemented for a diverse range of benign and malignant intracavitary lesions across two disciplines, with favorable perioperative outcomes and adherence to established oncologic principles. These findings parallel prior reports demonstrating the feasibility of MIS and robotic-assisted resection in carefully selected pediatric patients.

Since the landmark report in 1995 demonstrating reduced morbidity with MIS, multiple studies have established its role in resection of intracavitary lesions including cancer in children [1, 5, 14, 19]. While most previous series have focused primarily on solid tumors, our cohort included both cystic and solid masses, reflecting the spectrum of pathology encountered in contemporary pediatric surgery and urology practice when pathology is not predetermined. Robotic-assisted resection in this series was associated with minimal intraoperative blood loss and a low conversion rate, underscoring technical feasibility. Notably, both abdominal and thoracic procedures were successfully performed in children as young as 3 years of age and weighing 11.2 kg. This experience shows the applicability of robotic surgery for younger patients with careful selection and surgeon experience.

Indeed, smaller children present distinct technical challenges for robotic-assisted surgery. Trocar size relative to body wall thickness increases the potential for arm collisions, and limited working space constrains instrument range of motion compared with older children or adults. Nevertheless, the use of robotic platforms in smaller patients has expanded steadily. A systematic review including more than 1,400 children weighing less than 10 kg demonstrated the safety and feasibility of robotic procedures across diverse operations [20]. Although our series did not include patients under 10 kg, six children weighed between 10 and 17 kg, and all but one, whose postoperative course was influenced by relapsed malignancy, experienced no surgical complications. Importantly, we did not encounter any significant vascular injury. These findings further support the feasibility of robotic resection in carefully selected smaller children.

Patient selection for robotic-assisted resection evolved throughout the study period. Early experience was primarily limited to cystic lesions and well-circumscribed masses with favorable dissection planes. As institutional experience increased, indications expanded to include increasingly complex oncologic resections, organ-preserving procedures, and hepatobiliary operations. This progression was supported by multidisciplinary imaging review, growing surgeon experience, and collaboration with adult surgical oncology, hepatobiliary, colorectal, thoracic, and urologic specialists when advanced resection and reconstruction were anticipated. Yet, extensive vascular involvement, inability to achieve an oncologically sound resection robotically, and anticipated need for major vascular reconstruction remain relative contraindications.

Robotic-assisted surgery may also confer meaningful perioperative advantages. Previous studies of open abdominal and thoracic resections have reported greater postoperative opioid requirements and hospital stays averaging 4–5 days [21]. Even with opioid-reduction initiatives, reported morphine equivalent dosing remains higher than that observed in our cohort [21, 22]. In contrast, our patients demonstrated low postoperative opioid utilization and abbreviated hospitalization, with two-thirds discharged on or before postoperative day one. Adequate pain control was confirmed through outpatient documentation and absence of additional opioid prescriptions. These reductions in pain burden and length of stay are particularly relevant in pediatric oncology, where timely resumption of adjuvant therapy may influence overall treatment continuity [14, 26].

Favorable outcomes in this cohort likely reflect not only patient selection but also a standardized perioperative framework. A consistent multidisciplinary process, including regional anesthesia, multimodal analgesia, early extubation, early mobilization, and prompt diet advancement, aligned with core principles of Enhanced Recovery After Surgery (ERAS) pathways [23–25]. Integration of these measures likely augmented the intrinsic advantages of minimally invasive robotic surgery, contributing to the abbreviated hospital stays and low opioid requirements observed in this series.

The role of robotic surgery for SPNs remains an area of ongoing investigation. While some prior data have suggested caution when applying minimally invasive approaches to SPNs due to the complexity of pancreatic resection and the potential consequences of tumor violation, more recent series have demonstrated the feasibility of robotic resection in carefully selected patients [3, 26, 27]. In our cohort, all three SPNs were successfully resected robotically with negative margins and without tumor spillage or recurrence. However, two out of four patients experienced major postoperative complications following pancreatic resection. These findings suggest that although robotic resection of SPN can be performed while maintaining oncologic principles, the morbidity associated with complex pancreatic operations remains substantial and should be considered during patient selection and operative planning.

Our center has a strong history of applying minimally invasive techniques to pediatric intracavitary cysts and neoplasms [14, 15, 28]. This experience has facilitated the adoption of robotic-assisted surgery at our institution [29]. The close collaboration between pediatric surgery and urology services has supported the upward trend of robotic cases in recent years. Several cases in this series were performed in close partnership between pediatric surgery and urology, particularly when organ preservation or complex reconstruction was required. For example, nephron-sparing resection for WT in a child with Beckwith–Wiedemann syndrome required coordinated operative planning to balance vascular control, oncologic clearance with maximal preservation of renal reserve, ureteral stent placement, and reconstruction of the renal pelvis. Such collaboration strengthens oncologic quality by leveraging complementary expertise in tumor biology, resection strategy, reconstruction, and long-term functional considerations. Importantly, this partnership fosters shared learning across service lines, allowing principles from pediatric surgery and urology to be applied collectively in complex cases. This multidisciplinary approach reinforces that the successful application of robotic technology in pediatric intracavitary lesions is grounded not only in technical capability, but also in integrated surgical judgment and collaborative decision-making.

Despite these advantages, concerns regarding the oncologic safety of minimally invasive and robotic surgery persist. Port-site recurrence, tumor dissemination, and tumor rupture have historically been cited as potential risks of minimally invasive oncologic surgery [5, 13]. Additionally, the lack of haptic feedback in robotic platforms raises theoretical concerns regarding capsular disruption and tumor spillage [9, 10]. However, contemporary pediatric series have demonstrated low rates of tumor spillage and recurrence when oncologic principles are maintained, including careful tumor handling, specimen containment, and avoidance of capsule violation [3, 11, 12, 28]. In our cohort, no intraoperative tumor spillage or port-site recurrence occurred, and all malignant lesions were resected with negative margins.

Minimally invasive approaches have demonstrated acceptable oncologic outcomes in children, including low local recurrence and relapse rates across thoracic and abdominal malignancies [14, 28]. Earlier studies support oncologic equivalence in carefully selected pediatric patients, including those with WT and other solid neoplasms. Further, patients having minimally invasive tumor resections were shown to remain more on time for their next therapy cycle than tumor volume-matched patients having open resections [6, 10, 13, 19, 30]. In our cohort, oncologic outcomes also were similarly favorable. Recurrence occurred in a single patient with stage III, group III bladder rhabdomyosarcoma, an aggressive malignancy associated with recurrence rates approaching one-third [31]. All remaining patients with malignant disease remained without evidence of relapse following robotic resection during the available follow-up period, suggesting that oncologic principles were maintained in appropriately selected cases.

This study is limited by its retrospective design and absence of a contemporaneous open and MIS control groups. The objective, however, was to characterize our institutional experience rather than establish comparative effectiveness. Future studies incorporating matched open or laparoscopic cohorts would better define relative benefits. Additionally, the number of malignant solid tumors resected robotically remains modest, although case volume has increased in recent years, reflecting growing institutional adoption. As experience accrues, larger analyses may permit more robust evaluation of oncologic durability and perioperative outcomes. Finally, as a single-center experience, these findings may not be generalizable to institutions with differing patient populations, technical expertise, robot availability, or perioperative infrastructure. Unmeasured confounders, including surgeon discretion, evolving protocols, and patient-specific factors, may also have influenced operative selection and outcomes.

## Conclusion

Robotic-assisted surgery serves as a valuable adjunct for complex resection of intracavitary lesions in children. When applied within a multidisciplinary framework, this approach appears feasible in carefully selected patients, pending larger and longer-term studies. Robotic platforms may facilitate shorter hospital stays, reduced postoperative opioid requirements, and accelerated recovery, advantages particularly meaningful for children with malignancy who require timely continuation of therapy. Our experience supports the safety and feasibility of robotic-assisted resection for both benign and malignant pediatric masses.

## Data Availability

No datasets were generated or analysed during the current study.
